# Prospective study found that peripheral lymph node sampling reduced the false-negative rate of sentinel lymph node biopsy for breast cancer

**DOI:** 10.1186/s40880-016-0099-x

**Published:** 2016-04-04

**Authors:** Chao Han, Ben Yang, Wen-Shu Zuo, Yan-Song Liu, Gang Zheng, Li Yang, Mei-Zhu Zheng

**Affiliations:** School of Medicine and Life Sciences, University of Jinan-Shandong Academy of Medical Sciences, Jinan, 250022 Shandong P.R. China; Department of Surgery, Shandong Breast Center of Prevention and Treatment, Shandong Cancer Hospital Affiliated to Shandong University, Shandong Academy of Medical Sciences, 440 Jiyan Road, Jinan, 250117 Shandong P.R. China

**Keywords:** Breast cancer, Sentinel lymph node biopsy, Peripheral lymph node, False-negative rate, Skip metastasis

## Abstract

**Background:**

Although sentinel lymph node biopsy (SLNB) can accurately predict the status of axillary lymph node (ALN) metastasis, the high false-negative rate (FNR) of SLNB is still the main obstacle for the treatment of patients who receive SLNB instead of ALN dissection (ALND). The purpose of this study was to evaluate the clinical significance of SLNB combined with peripheral lymph node (PLN) sampling for reducing the FNR for breast cancer and to discuss the effect of “skip metastasis” on the FNR of SLNB.

**Methods:**

At Shandong Cancer Hospital Affiliated to Shandong University between March 1, 2012 and June 30, 2015, the sentinel lymph nodes (SLNs) of 596 patients with breast cancer were examined using radiocolloids with blue dye tracer. First, the SLNs were removed; then, the area surrounding the original SLNs was selected, and the visible lymph nodes in a field of 3–5 cm in diameter around the center (i.e., PLNs) were removed, avoiding damage to the structure of the breast. Finally, ALND was performed. The SLNs, PLNs, and remaining ALNs underwent pathologic examination, and the relationship between them was analyzed.

**Results:**

The identification rate of SLNs in the 596 patients was 95.1% (567/596); the metastasis rate of ALNs was 33.7% (191/567); the FNR of pure SLNB was 9.9% (19/191); and after the SLNs and PLNs were eliminated, the FNR was 4.2% (8/191), which was significantly decreased compared with the FNR before removal of PLNs (*P* = 0.028). According to the detected number (N) of SLNs, the patients were divided into four groups of N = 1, 2, 3, and ≥4; the FNR in these groups was 19.6, 9.8, 7.3, and 2.3%, respectively. For the patients with ≤2 or ≤3 detected SLNs, the FNR after removal of PLNs was significantly decreased compared with that before removal of PLNs (N ≤ 2: 14.0% vs. 4.7%, *P* = 0.019; N ≤ 3: 12.2% vs. 4.7%, *P* = 0.021), whereas for patients with ≥4 detected SLNs, the decrease in FNR was not statistically significant (*P* = 1.000). In the entire cohorts, the “skip metastasis” rate was 2.5% (15/596); the FNR caused by “skip metastasis” was 2.1% (4/191).

**Conclusions:**

The FNR of SLNB was associated with the number of SLNs. For patients with ≤3 detected SLNs, PLN sampling can reduce the FNR of SLNB to an acceptable level of less than 5%. Because of the existence of the “skip metastasis” and distinct metastasis patterns, the FNR of SLNB cannot be completely eliminated.

## Background

Breast cancer is a common cancer in China; the age-standardized rate (ASR) of incidence is estimated to be 23.2/100,000, and the ASR of mortality is approximately 4.9/100,000 [1, 2]. To accurately stage breast cancer, it is necessary to know a patient’s axillary lymph node (ALN) status. ALN status is also one of the most important indicators for determining the prognosis of patients with breast cancer and guiding axillary treatment. Axillary lymph node dissection (ALND) is an important part of the surgical treatment of invasive breast cancer; moreover, ALND is the most accurate method of evaluating the status of ALN metastasis. Patients with negative ALN status do not benefit from ALND, which increases the incidence of postoperative complications such as lymphedema and sensory and motor dysfunction. Greater awareness of breast cancer prevention and new treatment technologies have resulted in a higher proportion of patients with early-stage breast cancer. The tumor load of the regional lymph nodes has also decreased [3, 4], making it possible to narrow the range of patients who undergo axillary surgery.

In the 1990s, sentinel lymph node biopsy (SLNB) emerged as a new surgical procedure replacing the ALND of breast cancer, and “axilla-conserving” guidance measures were implemented. For sentinel lymph node (SLN)-negative patients with breast cancer, large-scale clinical trials have confirmed that SLNB and ALND result in no significant difference in terms of disease-free survival, overall survival, and recurrence-free survival [5–7]; other studies have shown that SLNB can accurately predict the metastasis of ALNs [8, 9]. In principle, SLN-negative patients can forego ALND, but false-negative results are still the main concern of physicians as well as patients who undergo SLNB instead of ALND. In 2014, the American Society of Clinical Oncology reported six trials of SLNB; in these trials, the false-negative rate (FNR) was between 4.6% and 16.7% [10]. Kim et al. [9] performed a meta-analysis and concluded that the average FNR of SLNB was 8.4% (range, 0%–29%). The American Society of Breast Surgeons established a task force to suggest acceptable standards for SLNB. In 2000, the task force recommended that the identification rate for SLNB be 85% or higher and that the FNR be 5% or lower [11]. Other studies demonstrated that, with the increase of the number of SLNs, the FNR of SLNB significantly decreased [12–14]. We conducted a prospective study of 596 patients with breast cancer who received SLNB combined with peripheral lymph node (PLN) sampling. To reduce the FNR, we attempted to increase the number of resected lymph nodes within a reasonable surgical field. There are many reasons for false-negative SLNB results; the phenomenon of “skip metastasis” [15] inevitably causes false negatives. This study also discusses the influence of “skip metastasis” on the FNR of SLNB.

## Patients and methods

### Patient selection and clinical data

The inclusion criteria for patients in the present study were as follows: (1) breast cancer confirmed by fine needle aspiration or biopsy; (2) preoperative clinical examination and imaging examination confirmed that the cases were cN0; (3) no anti-cancer therapy received before surgery; (4) after the SLNs and PLNs were resected (whether or not there was metastasis), patients underwent level I combined with level II lymph node dissection or complete ALND (including levels I, II, and III lymph nodes). Given these inclusion criteria, 596 patients with breast cancer who were treated at Shandong Cancer Hospital Affiliated to Shandong University, Shandong Province, China, between March 3, 2012 and June 30, 2015, were included in this study. All patients were women, aged 26–77 years, with an average age of 48.7 years and a median age of 41 years. In 367 patients, the tumor was located in the upper outer quadrant; in 83 patients, in the lower outer quadrant; in 73 patients, in the upper inner quadrant; in 30 patients, in the lower inner quadrant; and in 43 patients, in the central area. Of all 596 patients, 502 underwent breast resection plus ALND, and 94 patients underwent breast-conserving surgery. This study was approved by the ethics committee of the Shandong Cancer Hospital Affiliated to Shandong University (201503047).

### Methods

#### SLN tracer method

Blue dye combined with radionuclide was used for all SLNBs. Six to 18 h preoperatively, patients were injected with ^99m^Tc–Sc-labeled sulfur colloid (Beijing Shihong Pharmaceutical Center, Beijing, China) into the subcutaneous tissue around the tumor or breast parenchyma. The injection volume was 18.5–37 MBq. The Gamma Detection System (Neoprobe 2000, Dublin, OH, USA) was used preoperatively to detect the distribution of hot spots in the axillary and internal mammary region, and the gamma count of the injected site was recorded. After anesthesia, 1–2 mL of 1% methylene blue (Jiangsu Jumpcan Pharmaceutical Co., Taixing, Jiangsu, China) was subcutaneously injected in a single point around the tumor or in the nipple areola; the surgery began 10–15 min later. During the operation, we generally found the blue-stained lymphatics on the lateral border of the pectoralis major muscle, through the blue-stained lymph tube. We separated and removed the blue-stained lymph nodes and recorded their value using the gamma probe. Finally, we detected the entire axillary area (including the blue-stained lymph nodes), searched for the entire ALN radioactive peak, and separated those lymph nodes with a radiation intensity greater than the lowest radioactivity of the blue-stained lymph nodes or higher than 10% of the radioactivity peak.

#### Operation steps and the definition of SLNs and PLNs

SLNs detected by dye and isotope were cut out, and the first SLN encountered was removed. To delineate the scope of the PLNs, we also removed the lymph nodes in a field of 3–5 cm in diameter around the SLNs. To avoid destroying the surrounding nerves and blood vessels, blunt separation was performed within the range of fat connective tissue to remove the visible lymph nodes. The operation steps were as follows: (1) the lymph nodes found through blue-stained lymphatics were classified as SLNs, whether the gamma detection value was high or low; (2) the lymph nodes with radiation intensity greater than 10% of the peak were also referred to as SLNs, regardless of whether they were blue-stained; and (3) the remaining lymph nodes were classified as the PLNs. After removing the PLNs and the SLNs, the complete ALND or level I combined with level II lymph node dissection was carried out according to the situation. We conducted a pathologic examination of the SLNs, PLNs, and levels I, II, and III lymph nodes.

### Statistical analysis

SPSS 19.0 software (IBM Co., Chicago, IL, USA) was used for all statistical analyses, and the results were evaluated per the University of Louisville SLNB technique criteria [16]. The identification rate = the number of SLNs identified/the number of patients who underwent SLNB × 100%; FNR = the number of false-negative cases/the number of true-positive plus false-negative cases × 100%; sensitivity = the number of true-positive cases/the number of true-positive plus false-negative cases × 100%; accuracy rate = the number of true-negative plus true-positive cases/the number of patients who underwent SLNB × 100%; and negative predictive value = the number of true-negative cases/the number of true-negative plus false-negative cases × 100%.

A Chi square test was used to compare the sample rate, as well as Fisher’s exact test with α = 0.05. The four-fold table exact test (Fisher’s exact test) was applied for univariate analysis.

## Results

### SLN and PLN metastasis

SLNs were detected in 567 out of 596 patients; the identification rate was 95.1% (567/596). For the 567 patients in whom SLNs were detected, the number of detected SLNs was 1–6, and a total of 1026 SLNs were dissected, with an average of 1.81 SLNs per patient (median, 2). From the surrounding tissues (including PLNs), 0–9 lymph nodes were detected; a total of 1281 PLNs were dissected, with an average of 2.26 lymph nodes per patient (median, 3). Of these 567 patients, 191 had ALN metastasis, and 376 had no ALN metastasis. According to the detection number (N) of SLNs, the patients were divided into four groups of N = 1, 2, 3, and ≥4 (Table 1). For the 567 patients who underwent SLNB, the FNR was 9.9% (19/191); the sensitivity was 90.1% (172/191); the accuracy rate was 96.6% (548/567); and the negative predictive value was 95.2% (376/395), as shown in Table 2. When SLNB was combined with PLN sampling, the FNR was 4.2% (8/191); the sensitivity was 95.8% (183/191); the accuracy rate was 98.6% (559/567); and the negative predictive value was 97.9% (376/384), as shown in Table 3. The difference in FNR before and after PLN sampling was statistically significant (*P* = 0.028; Table 4).

**Table 1:** Grouping based on the number of SLNs

ALN status	No. of patients	No. of SLNs			
		1	2	3	≥4
Negative ALNs	376	112	111	89	64
Positive ALNs					
SLN(+) and nSLN(+)	72	19	22	14	17
SLN(+) and nSLN(–)	100	18	33	24	25
SLN(–) and PLN(+)	11	7	3	1	0
SLN(–) and PLN(–)	8	2	3	2	1
Total	567	158	172	130	107

Abbreviations: *SLN* sentinel lymph node, *ALN* axillary lymph node, *nSLN* non-sentinel lymph node, *PLN* peripheral lymph node

**Table 2:** ALN metastasis state of 567 patients detected by SLNB

SLN status	ALN status		Total
	(+)	(–)	
(+)	172 (a)	0 (c)	172
(–)	19 (b)	376 (d)	395
Total	191	376	567

For SLNB, the FNR was $\left( {\rm{b}} \right)/\left( {{\rm{a + b}}} \right) = 19\;\left( {172{\rm{ + 19}}} \right) = 9.9\%$. Sensitivity was $\left( {\rm{a}} \right)\left/\,( {{\rm{a + b}}} \right) = 172\;\left( {172{\rm{ + 19}}} \right) = 90.1\%$. Accuracy rate was ${{\left( {{\text{a}}{\mathbf{ + }}{\text{d}}} \right)} \mathord{\left/ {\vphantom {{\left( {{\text{a}}{\mathbf{ + }}{\text{d}}} \right)} {\left( {{\text{a}}\text{ + }{\text{b}}{\mathbf{ + }}{\text{c}}{\mathbf{ + }}{\text{d}}} \right) = {{\left( {172\text{ + 376}} \right)} \mathord{\left/ {\vphantom {{\left( {172\text{ + 376}} \right)} {\left( {172{\mathbf{ + }}19{\mathbf{ + }}0{\mathbf{ + }}376} \right)}}} \right. \kern-0pt} {\left( {172{\mathbf{ + }}19{\mathbf{ + }}0{\mathbf{ + }}376} \right)}} = 96.6\% }}} \right. \kern-0pt} {\left( {{\text{a}}\text{ + }{\text{b}}{\mathbf{ + }}{\text{c}}{\mathbf{ + }}{\text{d}}} \right) = {{\left( {172\text{ + 376}} \right)} \mathord{\left/ {\vphantom {{\left( {172\text{ + 376}} \right)} {\left( {172{\mathbf{ + }}19{\mathbf{ + }}0{\mathbf{ + }}376} \right)}}} \right. \kern-0pt} {\left( {172{\mathbf{ + }}19{\mathbf{ + }}0{\mathbf{ + }}376} \right)}} = 96.6\% }}$. Negative predictive value was ${{\left( {\text{d}} \right)} \mathord{\left/ {\vphantom {{\left( {\text{d}} \right)} {\left( {{\text{b}}{\mathbf{ + }}{\text{d}}} \right)}}} \right. \kern-0pt} {\left( {{\text{b}}{\mathbf{ + }}{\text{d}}} \right)}} = {{376} \mathord{\left/ {\vphantom {{376} {\left( {19{\mathbf{ + }}376} \right)}}} \right. \kern-0pt} {\left( {19{\mathbf{ + }}376} \right)}} = 95.2\%$

Abbreviations: *ALN* axillary lymph node, *SLNB* sentinel lymph node biopsy, *SLN* sentinel lymph node, *FNR* false-negative rate

**Table 3:** ALN metastasis state of 567 patients detected with SLNB and PLN sampling

SLN + PLN status	ALN status		Total
	(+)	(–)	
(+)	183 (a′)	0 (c′)	183
(–)	8 (b′)	376 (d′)	384
Total	191	376	567

For SLNB + PLN sampling, the FNR was $\left( {{{\rm{b}}^\prime }} \right)/\left( {{{\rm{a}}^\prime }{\rm{ + }}{{\rm{b}}^\prime }} \right) = 8\;\left( {183{\rm{ + 8}}} \right) = 4.2\%$. Sensitivity was $\left( {{{\rm{a}}^\prime }} \right)/\left( {{{\rm{a}}^\prime }{\rm{ + }}{{\rm{b}}^\prime }} \right) = 183\;\left( {183{\rm{ + 8}}} \right) = 95.8\%$. Accuracy rate was ${{\left( {{\text{a}}^{{\mathbf{\prime }}} {\mathbf{ + }}{\text{d}}^{{\mathbf{\prime }}} } \right)} \mathord{\left/ {\vphantom {{\left( {{\text{a}}^{{\mathbf{\prime }}} {\mathbf{ + }}{\text{d}}^{{\mathbf{\prime }}} } \right)} {\left( {{\text{a}}^{{\mathbf{\prime }}} \text{ + }{\text{b}}^{{\mathbf{\prime }}} {\mathbf{ + }}{\text{c}}^{{\mathbf{\prime }}} {\mathbf{ + }}{\text{d}}^{{\mathbf{\prime }}} } \right) = {{\left( {183\text{ + 376}} \right)} \mathord{\left/ {\vphantom {{\left( {183\text{ + 376}} \right)} {\left( {183{\mathbf{ + 8 + }}0{\mathbf{ + }}376} \right)}}} \right. \kern-0pt} {\left( {183{\mathbf{ + 8 + }}0{\mathbf{ + }}376} \right)}} = 98.6\% }}} \right. \kern-0pt} {\left( {{\text{a}}^{{\mathbf{\prime }}} \text{ + }{\text{b}}^{{\mathbf{\prime }}} {\mathbf{ + }}{\text{c}}^{{\mathbf{\prime }}} {\mathbf{ + }}{\text{d}}^{{\mathbf{\prime }}} } \right) = {{\left( {183\text{ + 376}} \right)} \mathord{\left/ {\vphantom {{\left( {183\text{ + 376}} \right)} {\left( {183{\mathbf{ + 8 + }}0{\mathbf{ + }}376} \right)}}} \right. \kern-0pt} {\left( {183{{ + 8 + }}0{\mathbf{ + }}376} \right)}} = 98.6\% }}$. Negative predictive value was ${{\left( {{\text{d}}^{{\mathbf{\prime }}} } \right)} \mathord{\left/ {\vphantom {{\left( {{\text{d}}^{{\mathbf{\prime }}} } \right)} {\left( {{\text{b}}^{{\mathbf{\prime }}} {\mathbf{ + }}{\text{d}}^{{\mathbf{\prime }}} } \right)}}} \right. \kern-0pt} {\left( {{\text{b}}^{{\mathbf{\prime }}} {\mathbf{ + }}{\text{d}}^{{\mathbf{\prime }}} } \right)}} = {{376} \mathord{\left/ {\vphantom {{376} {\left( {{{8 + }}376} \right)}}} \right. \kern-0pt} {\left( {{{8 + }}376} \right)}} = 97.9\%$

Abbreviations: *ALN* axillary lymph node, *SLNB* sentinel lymph node biopsy, *PLN* peripheral lymph node, *SLN* sentinel lymph node, *FNR* false-negative rate

**Table 4:** FNR comparison before and after PLN sampling

No. of SLNs	No. of patients	FNR of SLNB (%)		χ^2^ value		*P* value
		Before PLN sampling	After PLN sampling			
N = 1	158	19.6	4.3	5.059	0.024	
N = 2	172	9.8	4.9	0.480	0.488	
N = 3	130	7.3	4.9	<0.001	1.000	
N ≥ 4	107	2.3	2.3	<0.001	1.000	
Total	567	9.9	4.2	4.822	0.028	

Abbreviations: *FNR* false-negative rate, *PLN* peripheral lymph node, *SLNB* sentinel lymph node biopsy, *SLN* sentinel lymph node, *N* number of SLNs

### The association between the number of detected SLNs and FNR

The FNR observed in the N = 1, 2, 3, and ≥4 groups was 19.6, 9.8, 7.3, and 2.3%, respectively (Table 4); the difference was statistically significant (χ^2^ = 7.856, *P* = 0.049). The FNR for pure SLNB was higher than 5% in the N = 1, 2, and 3 groups. When SLNB was combined with PNR sampling, the FNR was 14.0% for the N ≤ 2 group. After PLNs were removed, the FNR decreased to 4.7%; this reduction in the FNR was statistically significant (χ^2^ = 5.515, *P* = 0.019). For the N ≤ 3 group, the FNR was 12.2%. After PLNs were removed, the FNR decreased to 4.7%; this reduction was also statistically significant (χ^2^ = 5.286, *P* = 0.021). No significant FNR decrease was observed in the N ≥ 4 group (*P* = 1.000; Table 4).

### The association between “skip metastasis” and FNR

In this study, 15 patients had “skip metastasis.” Among these 15 patients, 3 had level I (−), II (+), and III (−); 1 had level I (−), II (−), and III (+); and 11 had level I (+), II (−), and III (+). The occurrence rate of “skip metastasis” was 2.5% (15/596). After SLNB was combined with PLN sampling, eight patients had false-negative results; among these eight patients, four had “skip metastasis.” The FNR caused by “skip metastasis” accounted for 2.1% (4/191) of cases.

## Discussion

In the present study, the FNR of SLNB was 9.9% (19/191), which is within an average level. The FNR was significantly reduced to 4.2% (8/191) after SLNB was combined with PLN sampling (*P* = 0.028). According to the grouping by the number of detected SLNs, for the patients with ≤2 or ≤3 detected SLNs, the FNR after removal of PLNs was significantly decreased compared with that before removal of PLNs (*P* = 0.019 and 0.021, respectively) and reached an acceptable level of less than 5%. For the patients with ≥4 detected SLNs, after the PLNs were removed, the FNR did not change (2.3% vs. 2.3%, *P* = 1.000). Our previous results showed that for patients with ≥4 detected SLNs, the FNR of SLNB was 1.9%, and the accuracy rate was 98.7% [14]. Thus, we conclude that SLNB can predict the state of ALNs, and it is not necessary to carry out PLN sampling for patients with ≥4 detected SLNs. The FNR caused by “skip metastasis” in this study accounted for 2.1% (4/191); “skip metastasis” will result in false negative results of SLNB, which cannot be completely eliminated.

Many researchers have confirmed the association between the number of detected SLNs and the FNR of SLNB. The American College of Surgeons Oncology Group Z1071 trial found that for patients with 2 and ≥3 detected SLNs, the FNR of SLNB was 21.1% and 9.1%, respectively (*P* = 0.007) [13]. Yi et al. [17] found that when the number of SLNs reached 5, generally more than 99% positive SLNs could be found, which is similar to our finding. We found that, with an increased number of SLNs, the FNR of SLNB decreased (*P* = 0.049); but after grouping patients based on the number of detected SLNs, the FNR of SLNB could be reduced to less than 5% by PLN sampling for those with ≤3 detected SLNs.

Most breast cancers follow the metastasis route of level I → level II → level III, and the metastasis of discontinuous pattern also exists in some patients. According to previous studies, the metastasis rate of the interpectoral nodes was 2.6% to 25.9% [18, 19], whereas the incidence of “skip metastasis” was between 1.5% and 19.2% [20–22]. If SLNB was performed on patients with “skip metastasis,” false-negative results may have also occurred [22, 23]. Breast cancer metastases may also first invade the internal mammary lymph nodes (IMLNs). In the mid-20th century, the study of radical mastectomy in breast cancer showed that the overall metastasis rate of IMLNs was 18% to 33%; most patients had ALN metastasis, and pure IMLN metastasis was 2% to 11% [24–26]. If metastatic tumor cells move through the IMLN chain or “skip metastasis” jumps over axillary level I, or directly metastasizes to level II and III of lymph nodes, a conventional SLNB will produce a false-negative result. Considering current SLNB technology and the route of breast cancer lymphatic metastasis, we conclude that false-negative results of SLNB cannot be completely eliminated. Regardless of the methods performed (such as increasing the number of detected SLNs) to reduce the FNR of SLNB, because of the “skip metastasis” and distinct metastasis patterns of the lymph nodes in patients with breast cancer, there is a threshold past which the FNR of SLNB will not further decrease. In our study, the incidence of “skip metastasis” was 2.5% (15/596), which is at a “low level” of the normal range as reported in the literature; this is because only cN0 patients were enrolled in our study. Of the eight patients who had false-negative results after SLNB was combined with PLN sampling, “skip metastasis” occurred in four patients, and the FNR caused by “skip metastasis” accounted for only 2.1% (4/191). If the FNR is at a low level (less than 5%), SLNB is a reasonable alternative to ALND. Despite “skip metastasis” and distinct metastasis patterns, the resulting FNR is still in the acceptable range of less than 5%.

The present study had several limitations. First, PLN sampling performed in the area of a 3- to 5-cm diameter around the first traced SLN was only a clinical experiment to consider the lymphatic distribution in breast tissue. Currently, we have no proven parameters to explain this distance, and the anatomy of the lymph drainage route in this area needs to be studied further. Second, the sample size of an individual group was small after patients were grouped based on the number of detected SLNs, so a large study sample is needed for further validation.

Treatment of breast cancer has changed considerably since the beginning of the 21st century. From the largest tolerable treatment to the most minimally effective treatment, the temporal treatment trend for the local ALNs of breast cancer patients has been towards less use of axillary dissection [27]. Removing the PLNs located within a certain distance around the SLNs can effectively reduce the FNR of SLNB, which can enhance the understanding of SLNs and support the use of the SLNB combined with PLN sampling to decrease the FNR. Nevertheless, given the current SLNB technology and the phenomenon of “skip metastasis,” we recognize that the FNR of SLNB will not decrease to zero. Thus, using a combination of pathologic and biological tumor characteristics as predictors of ALN status could become another trend [27]. Future studies should include patient follow-up and prognostic evaluation.
